# Prognostic significance of IgG-Kappa positivity detected by serum immunofixation during daratumumab therapy in multiple myeloma

**DOI:** 10.48101/ujms.v131.14932

**Published:** 2026-11-03

**Authors:** Esra Cengiz, Ekin Kırcalı, Rıfat Furkan Aydın, Murat Çınarsoy

**Affiliations:** a Ankara Medicana International Hospital, Dept. of Hematology, Ankara, Türkiye;; b Ankara Education and Training Hospital, Dept. of Internal Medicine, Ankara, Türkiye;; c Balıkesir Atatürk City Hospital, Dept. of Hematology, Balıkesir, Türkiye

**Keywords:** Monoclonal antibody interference, minimal residual disease, treatment response, progression-free survival, overall survival, M-protein, CD38, paraprotein

## Abstract

**Background:**

Daratumumab, an immunoglobulin G kappa (IgG-κ) monoclonal antibody, can co-migrate with endogenous M-protein on serum immunofixation (IF), producing IgG-κ bands that may be misinterpreted as residual disease. We therefore evaluated treatment responses and the prognostic impact of daratumumab-associated IgG-κ IF positivity on progression-free survival (PFS) and overall survival (OS) in multiple myeloma (MM).

**Methods:**

We retrospectively analyzed 108 patients with MM treated with daratumumab-containing regimens at any line of therapy between January 2014 and January 2024. Serum IF IgG-κ positivity was assessed both at the first response assessment and during continued treatment. Treatment responses were classified according to the International Myeloma Working Group (IMWG) criteria, and PFS and OS were analyzed using the Kaplan–Meier method, the log-rank test, and multivariable Cox proportional hazards regression.

**Results:**

IgG-κ IF positivity was detected in 85 patients (78.7%) at first response, and the overall response rate to daratumumab was 64.8%. Patients positive at first response had longer PFS (14.0 vs. 7.0 months; *P* = 0.048) and higher very good partial response/complete response rates (65.9% vs. 43.5%; *P* = 0.058). In non–IgG-κ myeloma, treatment-emergent positivity was associated with a markedly higher deep-response rate (88.5% vs. 44.2%; *P* < 0.001) and a reduced risk of progression (hazard ratio 0.17; 95% confidence interval 0.03–0.74; *P* = 0.018). On multivariable analysis, older age independently predicted mortality, whereas persistent IF positivity did not worsen survival.

**Conclusions:**

IgG-κ IF positivity during daratumumab therapy should not be interpreted as residual disease in isolation; a substantial proportion likely reflects therapeutic antibody interference. Recognizing this interference is important to avoid overestimating residual disease and misclassifying treatment response, and confirmatory assays should be used where available.

## Introduction

Monoclonal antibodies are crucial for treating multiple myeloma (MM) (1). Daratumumab, a human immunoglobulin G kappa (IgG-κ) monoclonal antibody, exerts its antimyeloma activity by binding with high affinity to the CD38 epitope expressed on plasma cells, inducing plasma cell death through multiple mechanisms, including complement-dependent cytotoxicity, antibody-dependent cellular cytotoxicity, and antibody-dependent cellular phagocytosis and apoptosis (2).

Because daratumumab is an IgG1-kappa human monoclonal antibody with a molecular weight similar to that of the IgG-kappa M-protein secreted by clonal plasma cells in MM, it cannot be readily distinguished from endogenous monoclonal proteins on serum immunofixation electrophoresis (sIFE) or serum protein electrophoresis (SPEP) (3).

This analytical interference may result in false-positive findings during treatment response assessment, particularly in patients with IgG-kappa MM, and may complicate the interpretation of complete response (CR) according to the International Myeloma Working Group (IMWG) response criteria (4). Several approaches have been developed to overcome daratumumab interference, including the Hydrashift/daratumumab IFE reflex assay (DIRA), anti-daratumumab antibodies, and mass spectrometry-based techniques (5–8). However, these methods are available only in selected centers, and treatment response in routine clinical practice is still largely evaluated using conventional IF results. Some literature suggests that the emergence of an IgG-kappa band during treatment may represent therapeutic antibody interference rather than residual disease and could be associated with more favorable clinical outcomes (9). In this study, we aimed to evaluate treatment responses in patients with MM receiving daratumumab and to investigate the impact of IgG-kappa positivity detected by serum IF on progression-free survival (PFS) and overall survival (OS).

## Materials and methods

### Study design

This retrospective multicenter study included 108 patients diagnosed with MM who received daratumumab in combination with any treatment regimen at any line of therapy between January 2014 and January 2024. Patient demographics, cytogenetic characteristics at diagnosis, and baseline M-protein subtype were recorded. The presence of IgG-kappa positivity on sIFE was evaluated both at the first response assessment following daratumumab initiation and during subsequent treatment. The first response assessment was defined as the first scheduled evaluation of treatment response, performed approximately 1 month after the initiation of daratumumab-containing therapy, in accordance with institutional protocols. Treatment responses were assessed according to the IMWG response criteria.

PFS was defined as the time from the initiation of daratumumab therapy to disease progression or relapse. OS was defined as the time from diagnosis to death from any cause or the date of last follow-up.

### Inclusion criteria

Adult patients (≥18 years of age) with a diagnosis of MM who received daratumumab-containing therapy at any stage of treatment were included in the study.

### Exclusion criteria

Patients diagnosed with amyloidosis and those lacking available sIFE data were excluded from the study.

### Statistical analysis

Categorical variables were compared using the chi-square test, and continuous variables were analyzed using the Mann–Whitney U test. Survival curves were estimated using the Kaplan–Meier method and compared using the log-rank test. Multivariable Cox proportional hazards regression analysis was performed to adjust for age and other potential confounding factors. A two-sided *P*-value of < 0.05 was considered statistically significant. Statistical analyses were performed using R software (version 4.5.0; R Foundation for Statistical Computing, Vienna, Austria).

### Ethics approval

This multicenter study was approved by the local ethics committee (Ethics Committee Approval No. HRÜ/25.02.51). The study was performed in line with the principles of the Declaration of Helsinki.

## Results

A total of 108 patients who received daratumumab in any combination and at any line of therapy for MM were included in the study (Fig. 1). The median age at diagnosis was 58.4 years (range, 36.7–82.7); 66 (61.1%) patients were male and 42 (38.9%) were female. The most frequent myeloma subtype was IgG-kappa (36.1%), followed by IgG-lambda (20.4%), free kappa light chain (14.8%), free lambda light chain (10.2%), IgA-kappa (10.2%), and IgA-lambda (7.4%) subtypes. IgD-lambda, a rare subtype, was present in only one patient (0.9%) (Supplementary Fig. S1). High-risk cytogenetic features were present in 19.4% of the patients, whereas 80.6% belonged to the standard-risk group. The detailed patient demographics and baseline characteristics are presented in Table 1.

**Figure 1:**
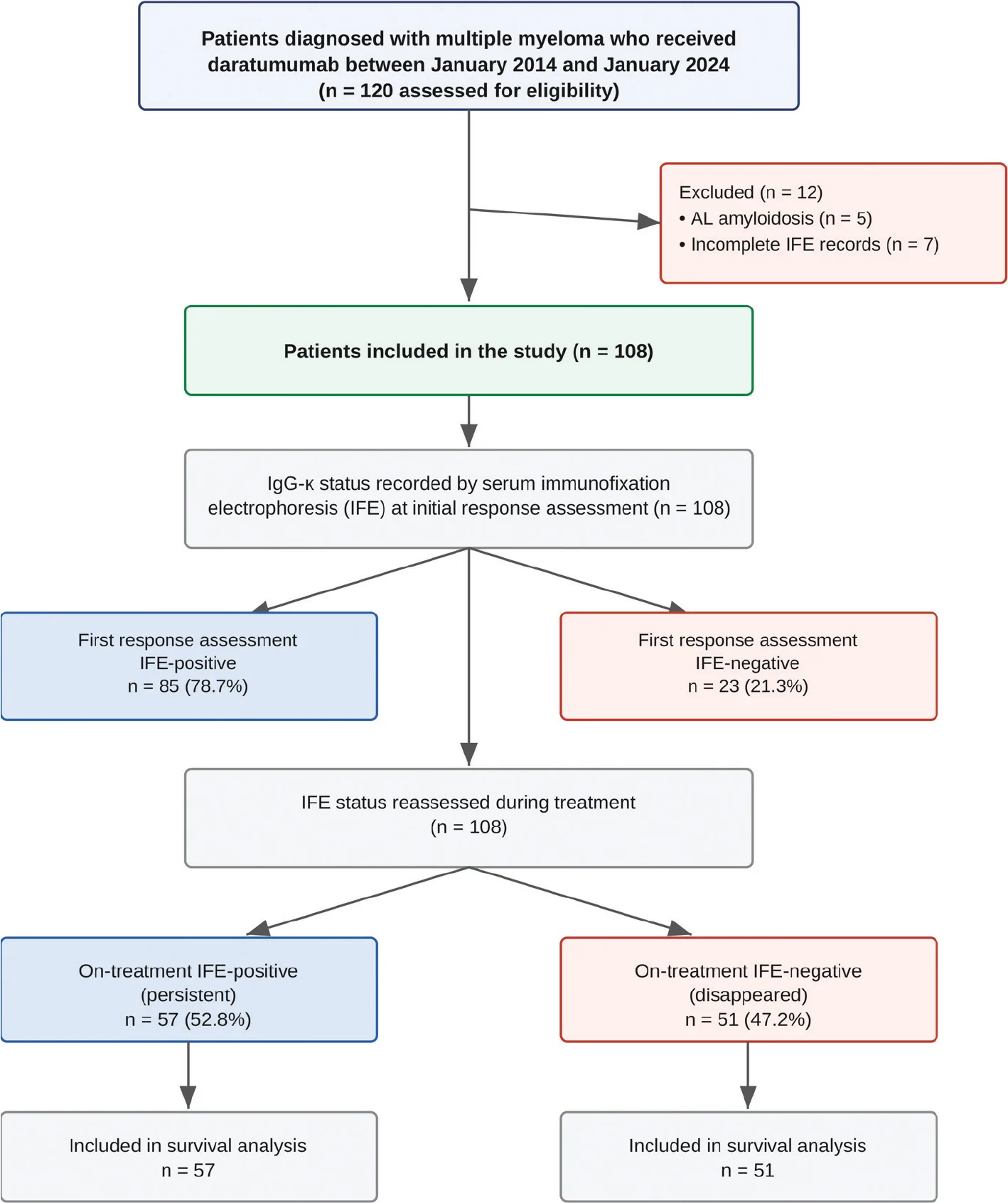
Patient selection and group allocation: STROBE flow diagram. AL: amyloid light chain; IFE: immunofixation electrophoresis. Figure 1 was prepared using a generative artificial intelligence-assisted analytical workflow applied to the underlying data.

**Table 1:** Baseline demographic and disease characteristics of the study population.

Characteristic	Overall cohort (*n* = 108)
Age at diagnosis, median (range)	58.4 (36.7–82.7)
Sex, male/female	66 (61.1%)/42 (38.9%)
**Myeloma subtype**	
IgG-kappa	39 (36.1%)
IgG-lambda	22 (20.4%)
Free kappa light chain	16 (14.8%)
Free lambda light chain	11 (10.2%)
IgA-kappa	11 (10.2%)
IgA-lambda	8 (7.4%)
IgD-lambda	1 (0.9%)
**Cytogenetic risk group**	
Standard risk	87 (80.6%)
High risk	21 (19.4%)
**Serum IFE IgG-κ at first response assessment**	
Positive	85 (78.7%)
Negative	23 (21.3%)
**Serum IFE IgG-κ during continued treatment**	
Positive	57 (52.8%)
Negative	51 (47.2%)
**Best response to daratumumab**	
Complete response (CR)	17 (15.7%)
Very good partial response (VGPR)	49 (45.4%)
Partial response (PR)	4 (3.7%)
Minimal response (MR)	1 (0.9%)
Progressive disease (PD)	37 (34.3%)
**Survival status**	
Alive	78 (72.2%)
Deceased	30 (27.8%)

Data are presented as *n* (%) unless otherwise specified.

IFE: immunofixation electrophoresis; IgG: immunoglobulin G; IgA: immunoglobulin A; IgD: immunoglobulin D; κ: kappa light chain; λ: lambda light chain; CR: complete response; VGPR: very good partial response; PR: partial response; MR: minimal response; PD: progressive disease.

At the first response assessment, serum IF was positive for IgG-kappa in 85 patients (78.7%) and negative in 23 patients (21.3%). During continued treatment, IgG-kappa IF positivity persisted in 57 patients (52.8%), whereas it disappeared in 51 patients (47.2%) (Supplementary Fig. S2). The overall response rate to daratumumab was 64.8% (70/108 patients). A very good partial response (VGPR) was achieved in 45.4% of patients, CR in 15.7%, and partial response (PR) in 3.7%. Progressive disease was observed in 34.3% of the patients (Supplementary Fig. S3). At the last visit, 78 patients (72.2%) were alive, and 30 (27.8%) had died.

Patients with IgG-kappa IF positivity at the first response assessment had a higher rate of deep response (VGPR or CR) (65.9% vs. 43.5%; *P* = 0.058; Supplementary Fig. S4). PFS was also longer in this group (14 vs. 7 months; *P* = 0.048; Fig. 2). In the Cox regression analysis, IgG-kappa positivity at first response showed a trend toward a reduced risk of progression (hazard ratio [HR] 0.52; 95% confidence interval [CI] 0.25–1.09; *P* = 0.078). No significant difference in OS was observed between the groups (*P* = 0.129; Supplementary Fig. S5).

**Figure 2:**
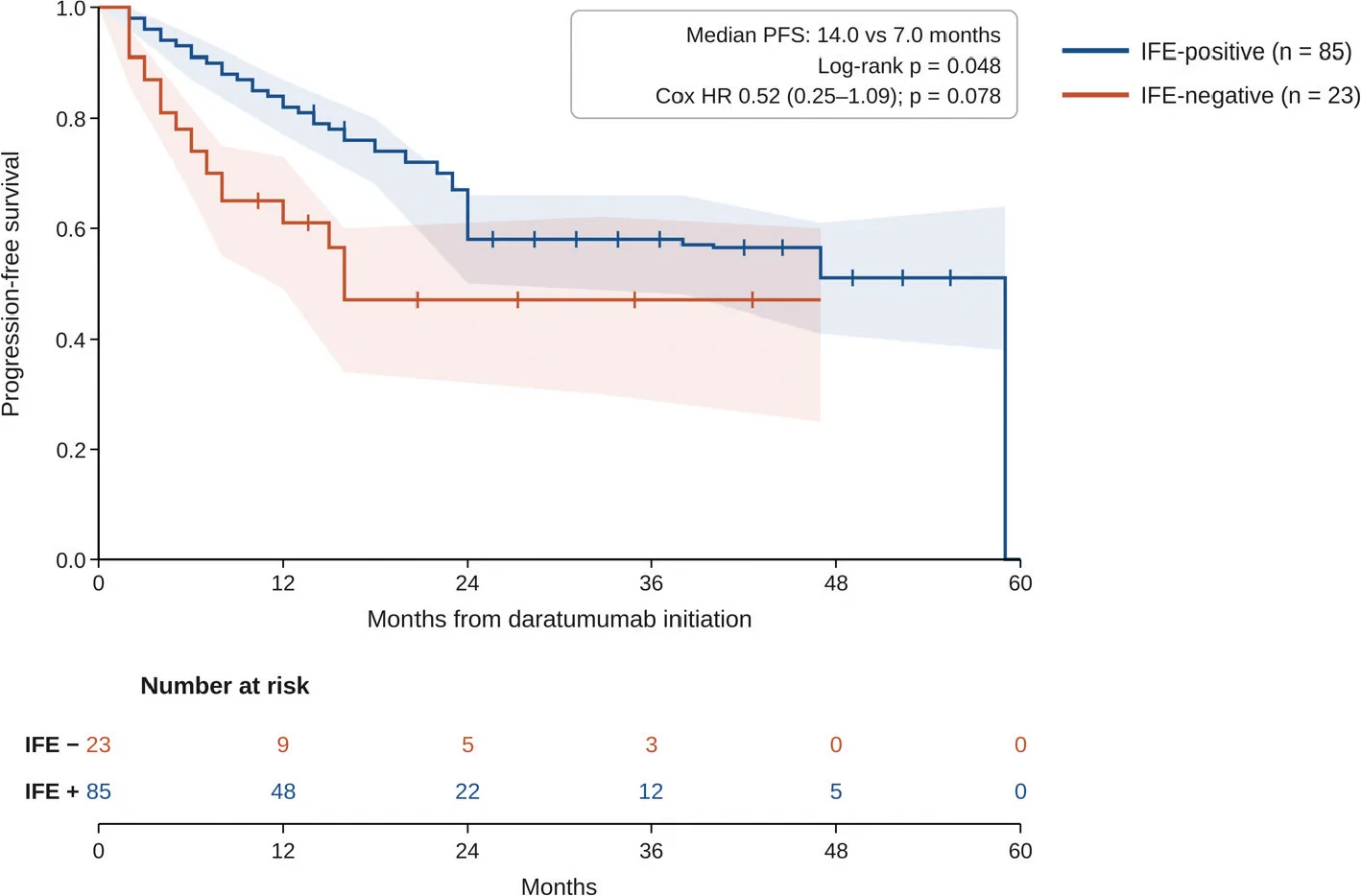
Progression-free survival according to the baseline IFE status: Kaplan–Meier curves. Tick marks denote censored observations; shaded areas represent 95% confidence intervals. PFS: progression-free survival; CI: confidence interval; HR: hazard ratio; IFE: immunofixation electrophoresis.

When patients with non–IgG-kappa myeloma were analyzed separately, the emergence of IgG-kappa IF positivity during treatment was found to be prognostically significant. Within this subgroup, patients with IgG-kappa light chain IF positivity demonstrated higher deep-response rates (68.1% vs. 45.5%). They also showed longer PFS (39.1 vs. 16.2 months; *P* = 0.13) and a trend toward longer OS (141.6 vs. 64 months; *P* = 0.05).

In the analysis based on IF findings during treatment, patients with persistent IgG-kappa IF positivity had higher deep-response rates (VGPR vs. CR: 70.2% vs. 51%; *P* = 0.065; Supplementary Fig. S6). In this group, the median PFS was not reached, whereas the median PFS in the IF-negative group was 39.1 months (*P* = 0.339; Supplementary Fig. S7). Although no significant difference in OS was observed, mortality was lower in the positive group (21.1% vs. 35.3%; Supplementary Fig. S8). Among patients with a non–IgG-kappa myeloma subtype at diagnosis, those with persistent IgG-kappa IF positivity during continued treatment had a significantly higher VGPR/CR rate (88.5% vs. 44.2%; *P* < 0.001; Fig. 3). PFS was also markedly longer, with the median PFS not reached, whereas the median PFS in the IF-negative group was 23.4 months (*P* = 0.01). In the Cox regression analysis, IgG-kappa IF positivity during continued treatment was associated with a significant reduction in the risk of progression (HR 0.17; 95% CI 0.03–0.74; *P* = 0.018). OS was longer in the IF-positive group (141.6 vs. 94.3 months; *P* = 0.113). When patients with IgG-kappa myeloma at diagnosis were analyzed separately, those whose IgG-kappa IF became negative during continued treatment showed longer OS (210.4 vs. 110.1 months; *P* = 0.027). Mortality was also lower in this group (12.5% vs. 29%).

**Figure 3:**
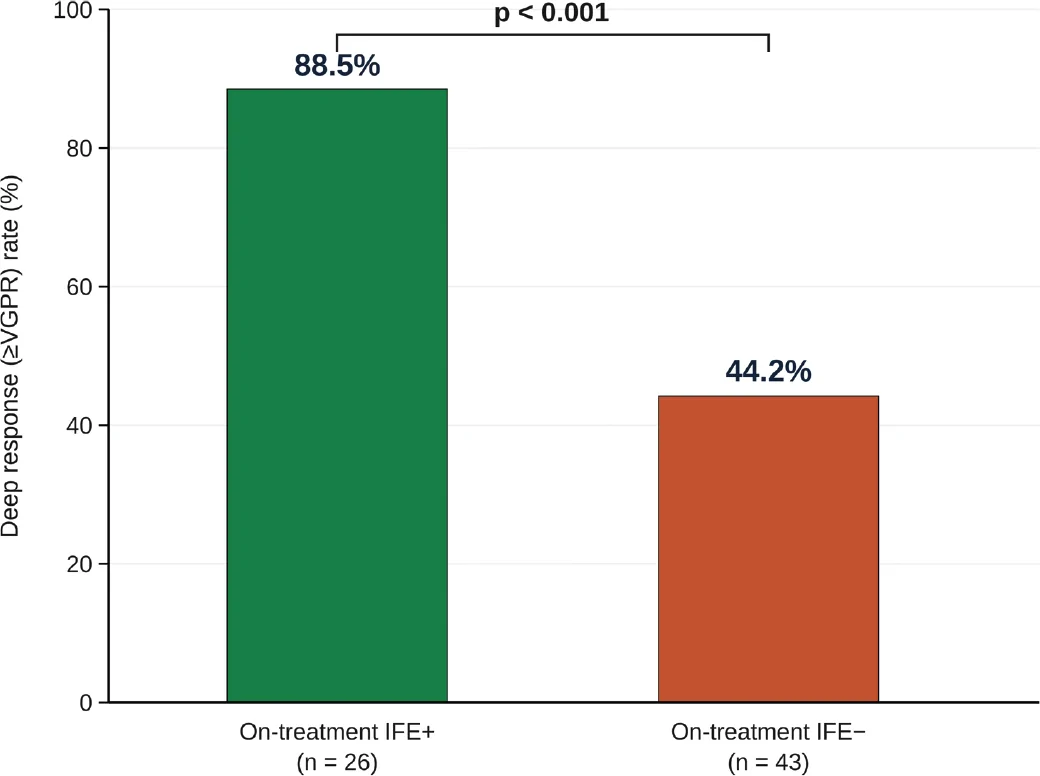
Deep-response rate in the non–IgG-κ subgroup stratified by on-treatment IFE status. The P-value was obtained using the χ² test. IFE: immunofixation electrophoresis; IgG-κ: immunoglobulin G kappa.

In analyses evaluating factors influencing mortality, high-risk cytogenetic features showed a trend toward an adverse prognostic effect on mortality (OR 2.36; *P* = 0.091). IgG-kappa IF positivity at first response (OR 0.40; *P* = 0.063) and IgG-kappa positivity during continued treatment (OR 0.49; *P* = 0.102) were associated with a trend toward lower mortality (Supplementary Fig. S9). In the multivariable Cox analysis, older age was identified as an independent risk factor for mortality (HR 1.07; *P* = 0.003), whereas IgG-kappa IF positivity during continued treatment was not associated with worse survival (Fig. 4).

**Figure 4:**
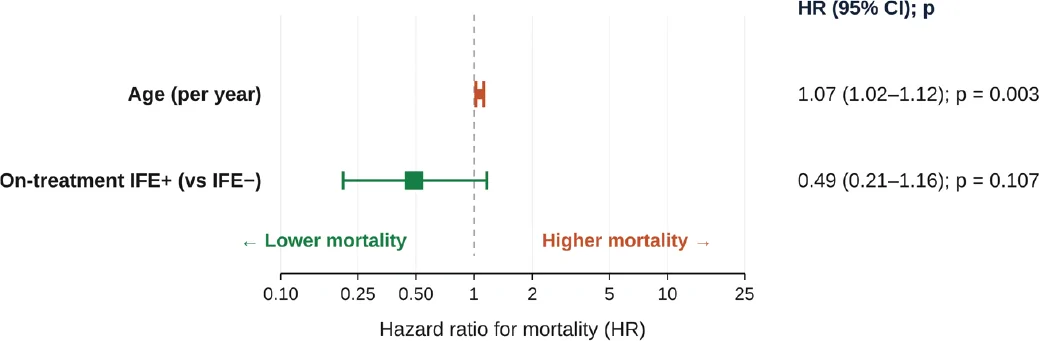
Multivariable Cox regression: independent determinants of mortality. Squares indicate the hazard ratio and horizontal lines the 95% confidence interval; the dashed vertical line marks HR = 1. Arrows indicate the direction of the effect: left, lower mortality; right, higher mortality. HR: hazard ratio; CI: confidence interval; IFE: immunofixation electrophoresis.

## Discussion

In this study, we evaluated the clinical significance of IgG-kappa positivity detected by sIFE in patients with MM receiving daratumumab-based therapy. The interference of anti-CD38 monoclonal antibodies with routine SPEP and immunofixation (IF) assays has long been recognized. Daratumumab migrates within the gamma region and may co-migrate with endogenous M-proteins, leading to false-positive results (5).

According to the IMWG response criteria, CR requires negative serum and urine immunofixation findings (4). Consequently, daratumumab-related interference has important clinical implications, particularly in CR assessment. Persistent detection of an IgG-kappa band during daratumumab therapy may result in false-positive immunofixation findings, even in patients who have achieved true biological remission. As a result, the interpretation of laboratory studies in patients receiving anti-CD38 therapy has become increasingly challenging.

Several approaches have been developed to overcome the interference of therapeutic mAbs. The Hydrashift and DIRA assays utilize anti-daratumumab antibodies to shift the therapeutic band to a different migration region, thereby facilitating the identification of residual monoclonal proteins (5, 6). In addition, matrix-assisted laser desorption/ionization time-of-flight (MALDI-TOF) mass spectrometry-based platforms, including MASS-FIX and the EXENT Analyzer, have demonstrated high sensitivity in distinguishing therapeutic antibodies from endogenous M-proteins. MASS-FIX has been reported to provide greater analytical sensitivity than conventional immunofixation and to detect low-level monoclonal proteins more accurately. However, these technologies remain available only in selected centers and have not yet been widely adopted in routine clinical practice because of their cost and infrastructure requirements (8, 10). Consequently, conventional immunofixation remains the primary method for response assessment at many institutions.

In addition to these antibody-specific reflex assays, bone marrow-based minimal residual disease (MRD) assessment has emerged as a highly sensitive method for evaluating treatment response in MM. The MRD evaluation using next-generation flow cytometry (NGF) or next-generation sequencing (NGS) can detect residual disease at a sensitivity of 10^–5^ to 10^–6^, and MRD negativity has been consistently associated with superior PFS and OS outcomes (11). Importantly, MRD assessment is independent of serum protein-based assays and is therefore not affected by therapeutic monoclonal antibody interference, including that caused by daratumumab. NGF-based approaches have been specifically adapted for use in patients receiving anti-CD38 therapy, overcoming the potential interference with plasma cell detection (12). As MRD-guided treatment strategies are increasingly being incorporated into clinical trials and are expected to enter routine clinical practice, they may offer a more practical and reliable complementary approach to response assessment in patients receiving anti-CD38 monoclonal antibody therapy.

The detection of an IgG-kappa band on IFE during daratumumab therapy should not be automatically interpreted as evidence of active disease. Instead, it may simply reflect the presence of therapeutic antibodies. This is particularly relevant in patients with non–IgG-kappa myeloma, in whom the emergence of a new IgG-kappa band is highly likely to represent daratumumab rather than residual disease. Consistent with this hypothesis, our study demonstrated that treatment-emergent IgG-kappa positivity in patients with non–IgG-kappa myeloma was not associated with adverse outcomes. On the contrary, these patients exhibited a tendency toward deeper responses, longer PFS, and improved OS. These findings are in agreement with the observations reported by Gigliotta et al., who showed that the appearance of an IgG-kappa band during anti-CD38 therapy in patients with non–IgG-kappa MM was associated with higher CR/VGPR rates and prolonged PFS (9). Collectively, these findings support the notion that a substantial proportion of IgG-kappa bands detected during treatment represent therapeutic daratumumab interference rather than persistent monoclonal disease.

We also observed that patients with IgG-kappa positivity at the first response assessment experienced longer PFS. Although this finding may initially appear paradoxical, it may serve as an indirect marker of adequate treatment. Previous studies have demonstrated a relationship between daratumumab pharmacokinetic exposure and clinical outcomes. Similarly, pharmacokinetic analyses from the GEN501 and SIRIUS trials demonstrated that greater daratumumab exposure correlated with prolonged PFS (13, 14). Therefore, persistent detection of an IgG-kappa band in the serum may indicate sustained therapeutic antibody exposure and effective target engagement.

In contrast, among patients with IgG-kappa myeloma at diagnosis, loss of IgG-kappa positivity during treatment was associated with superior OS. In this setting, disappearance of the IgG-kappa band may represent true eradication of the malignant paraprotein rather than clearance of therapeutic antibody interference. These findings suggest two distinct biological mechanisms. In patients with non-IgG-kappa myeloma, persistent IgG-kappa positivity is likely to reflect therapeutic antibody interference, whereas in patients with IgG-kappa myeloma, disappearance of the band may indicate genuine suppression of tumor-derived monoclonal protein production due to therapy. Accordingly, the interpretation of immunofixation results during anti-CD38 therapy requires consideration of both technical and biological factors.

This study has several limitations. Firstly, the IgG-kappa bands detected on immunofixation could not be directly confirmed as therapeutic daratumumab or residual disease-associated bands. Because Hydrashift, DIRA, and mass spectrometry-based confirmatory methods are not routinely available, our conclusions are based on indirect clinical associations. Secondly, the relatively limited sample size, particularly in subgroup analyses, may have reduced the statistical power of some comparisons. Nevertheless, this study has several important strengths, including its real-world design, separate evaluation of biologically distinct myeloma subtypes, and assessment of the prognostic significance of IF findings during ongoing treatment.

## Conclusion

In conclusion, our findings indicate that IgG-kappa positivity detected by IF during daratumumab therapy should not be interpreted as evidence of residual disease. In particular, among patients with non-IgG-kappa myeloma at diagnosis, treatment-emergent IgG-kappa positivity was associated with higher rates of deep response and longer PFS. These results highlight the clinical impact of therapeutic mAb interference on routine laboratory assessments and underscore the need for broader implementation of confirmatory techniques capable of distinguishing therapeutic antibodies from residual monoclonal proteins. Our results should be confirmed in further prospective, large-cohort trials.

## Supplementary Material



## Data Availability

The data supporting the findings of this study are available from the corresponding author upon reasonable request.
