# Supplementary material for: Prognostic significance of IgG-Kappa positivity detected by serum immunofixation during daratumumab therapy in multiple myeloma
Source: Ups J Med Sci. 2026 Nov 3;131:10.48101/ujms.v131.14932. doi: 10.48101/ujms.v131.14932 (PMC13737237; doi:10.48101/ujms.v131.14932)
Supplement: Supplementary file 1 [file UJMS-131-14932-s1.pdf]

Supplementary material

The following items are provided as supplementary material.

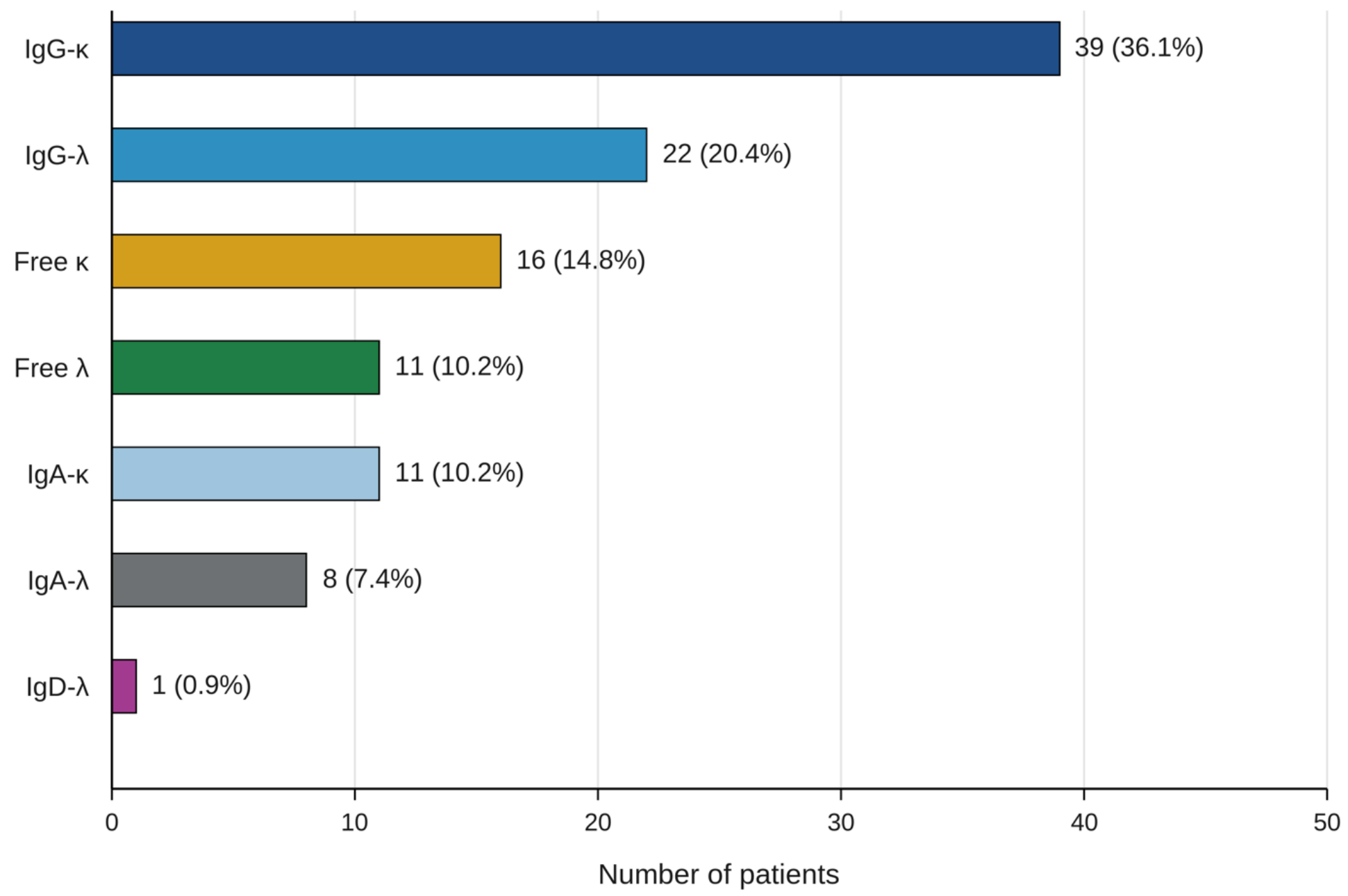

Supplementary Fig. S1. Distribution of myeloma subtypes (n = 108). IgG-κ, immunoglobulin G kappa; IgG-λ, immunoglobulin G lambda; IgA, immunoglobulin A; IgD, immunoglobulin D; κ, kappa light chain; λ, lambda light chain.

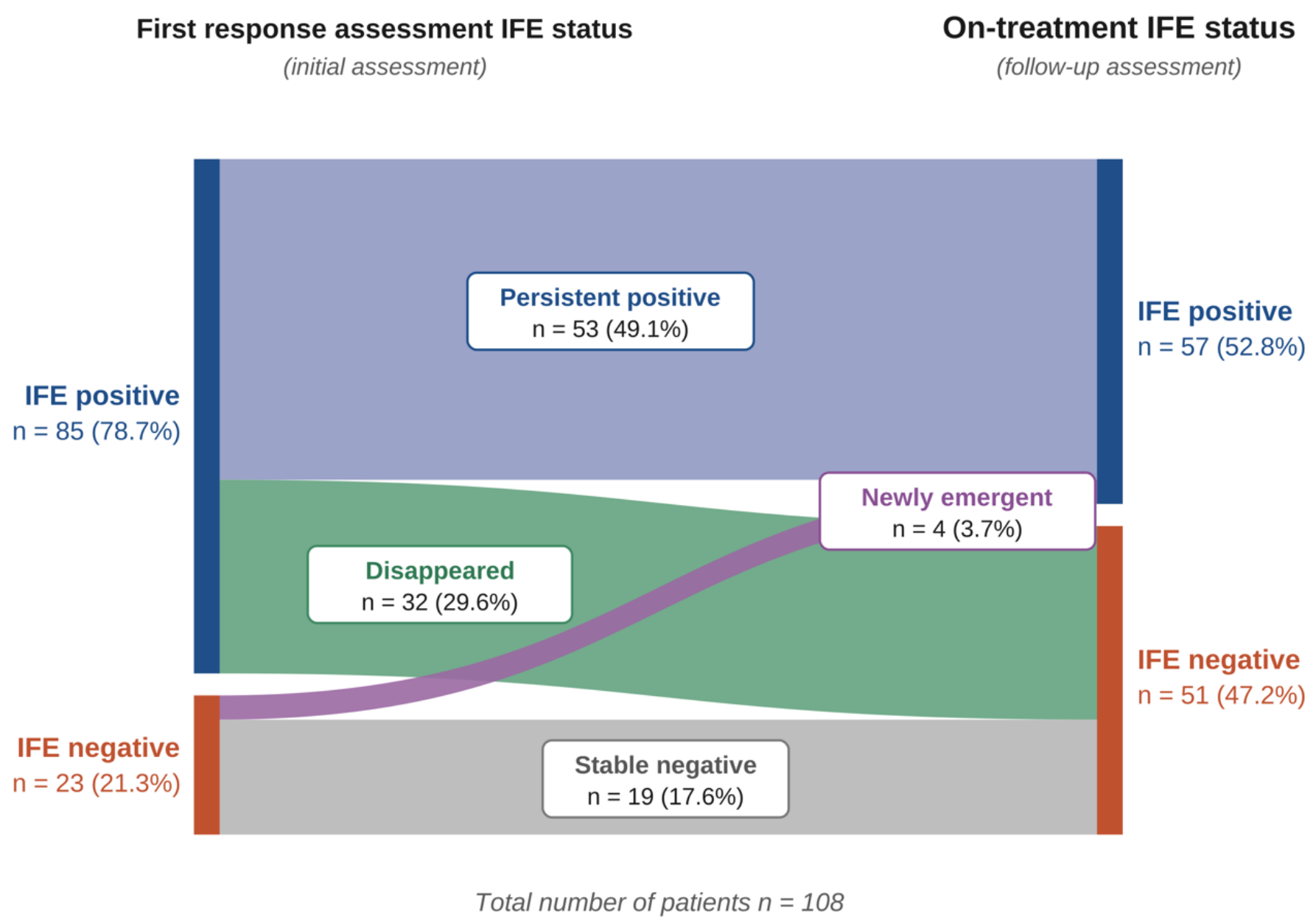

Supplementary Fig. S2. Transitions in serum IFE IgG-κ status during treatment (alluvial diagram). The bands link the baseline status (first response assessment) to the status during continued treatment. IFE, immunofixation electrophoresis.

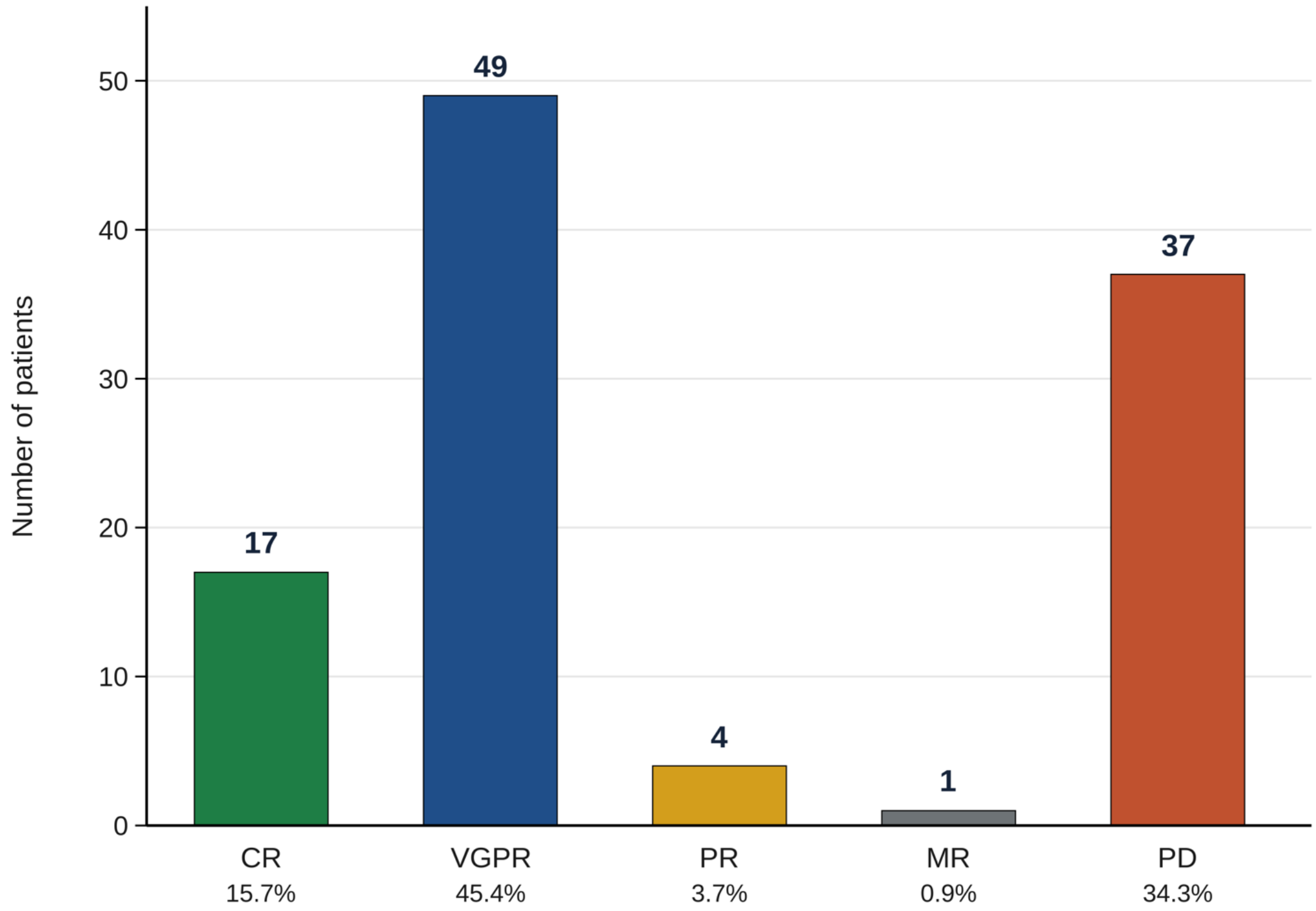

Supplementary Fig. S3. Distribution of the best response to daratumumab. CR, complete response; VGPR, very good partial response; PR, partial response; MR, minimal response; PD, progressive disease; ORR, overall response rate (≥PR).

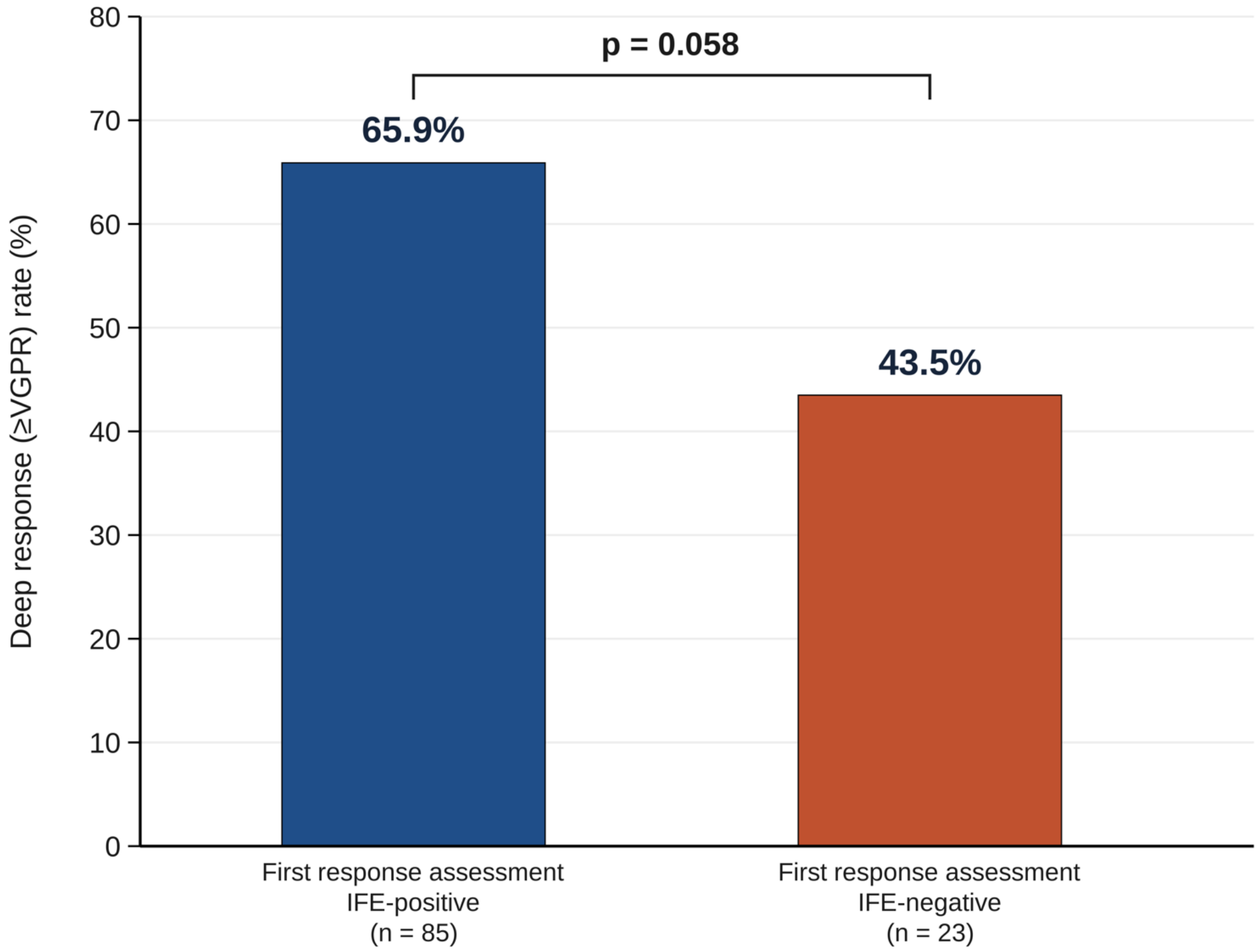

Supplementary Fig. S4. Deep response (≥VGPR) rate according to the baseline IFE status. Deep response was defined as the best treatment response of VGPR or CR. The p-value was obtained using the χ^2 test. IFE, immunofixation electrophoresis; CR, complete response; VGPR, very good partial response.

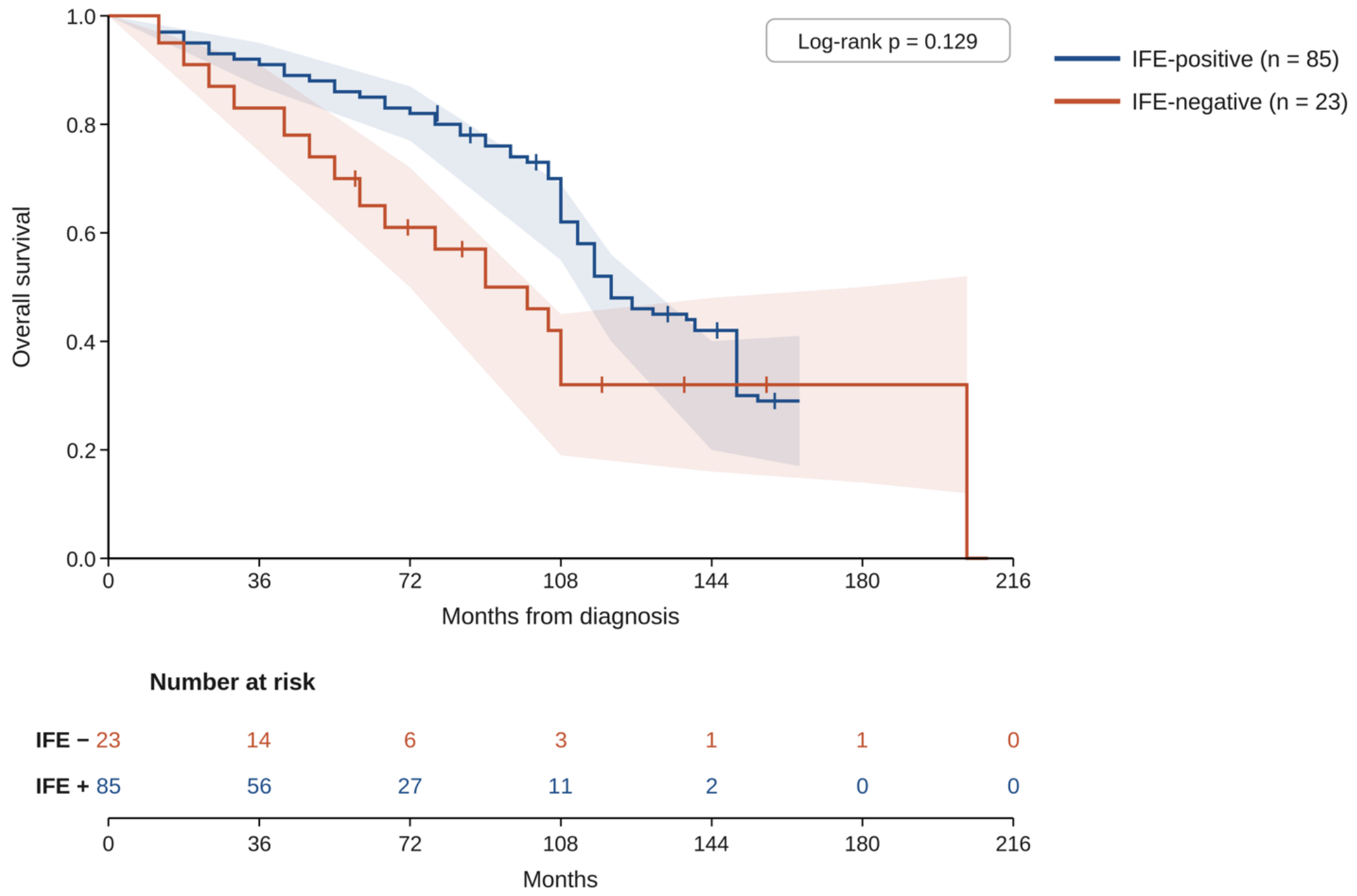

Supplementary Fig. S5. Overall survival according to the baseline IFE status: Kaplan–Meier curves. Overall survival was calculated from the date of diagnosis. Tick marks denote censored observations; shaded areas represent 95% confidence intervals. OS, overall survival; IFE, immunofixation electrophoresis.

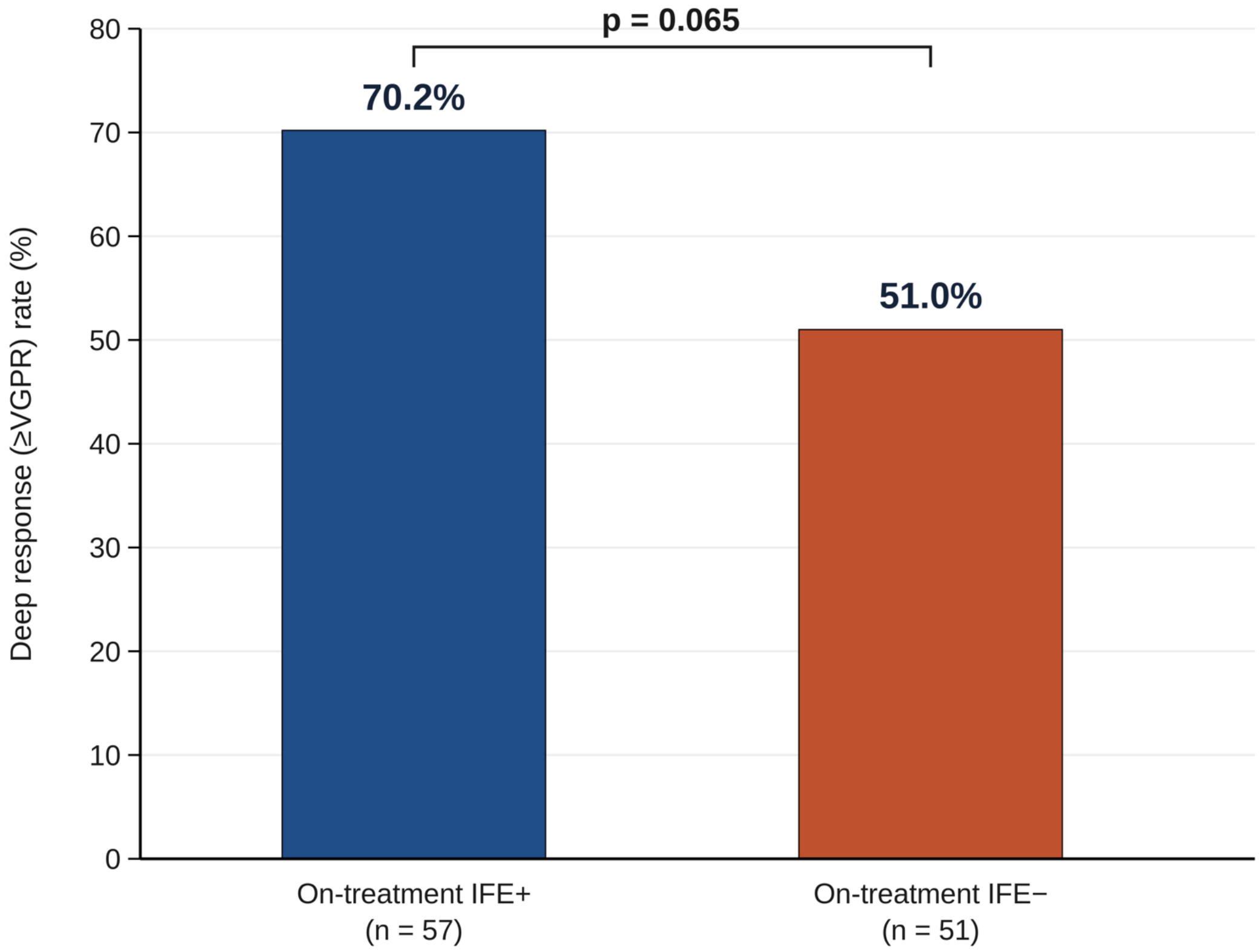

Supplementary Fig. S6. Deep response rate according to on-treatment IFE status (persistently positive vs. cleared). The p-value was obtained using the χ^2 test. IFE, immunofixation electrophoresis.

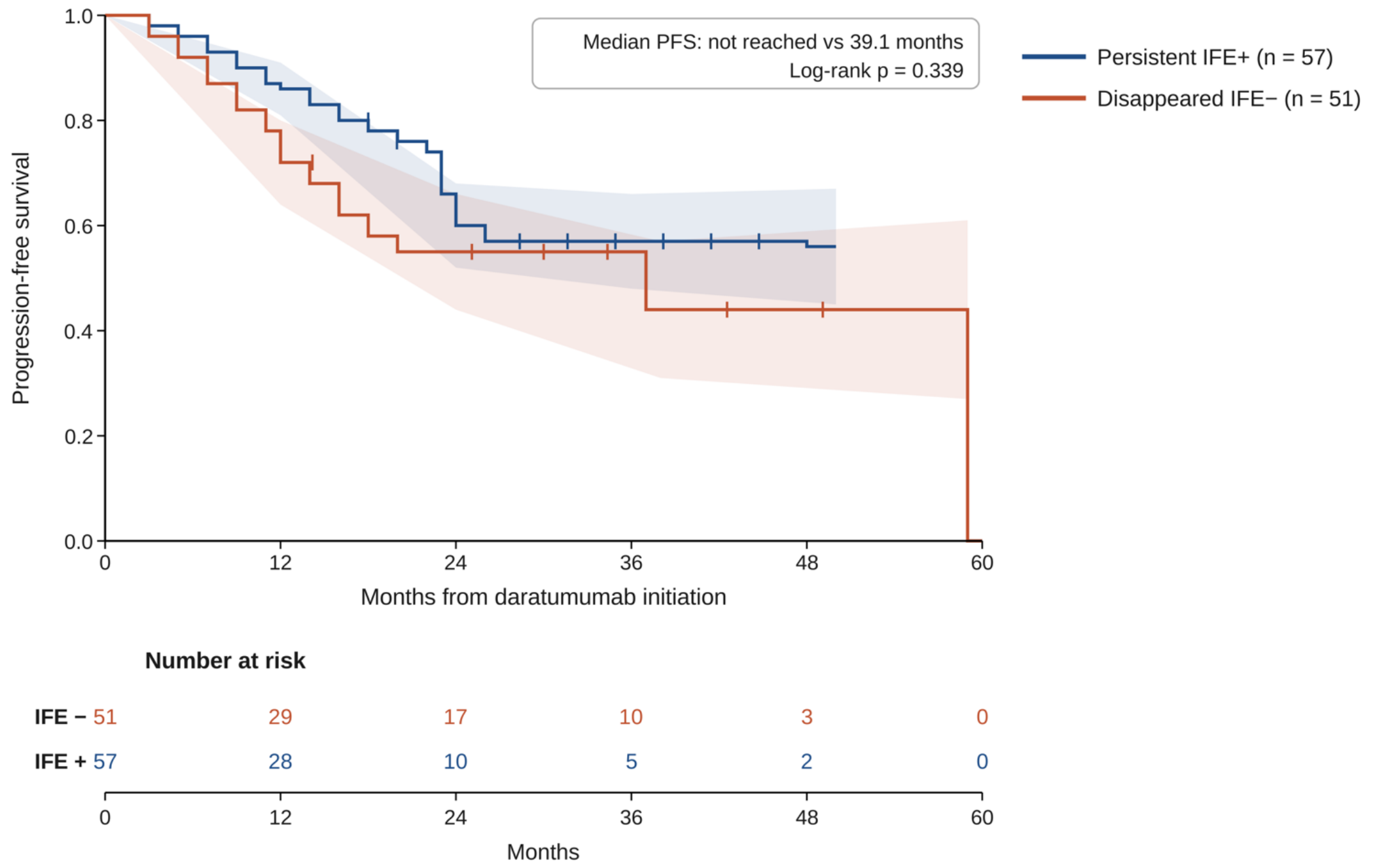

Supplementary Fig. S7. Progression-free survival according to on-treatment IFE status: Kaplan–Meier curves. Tick marks denote censored observations; shaded areas represent 95% confidence intervals. PFS, progression-free survival; IFE, immunofixation electrophoresis.

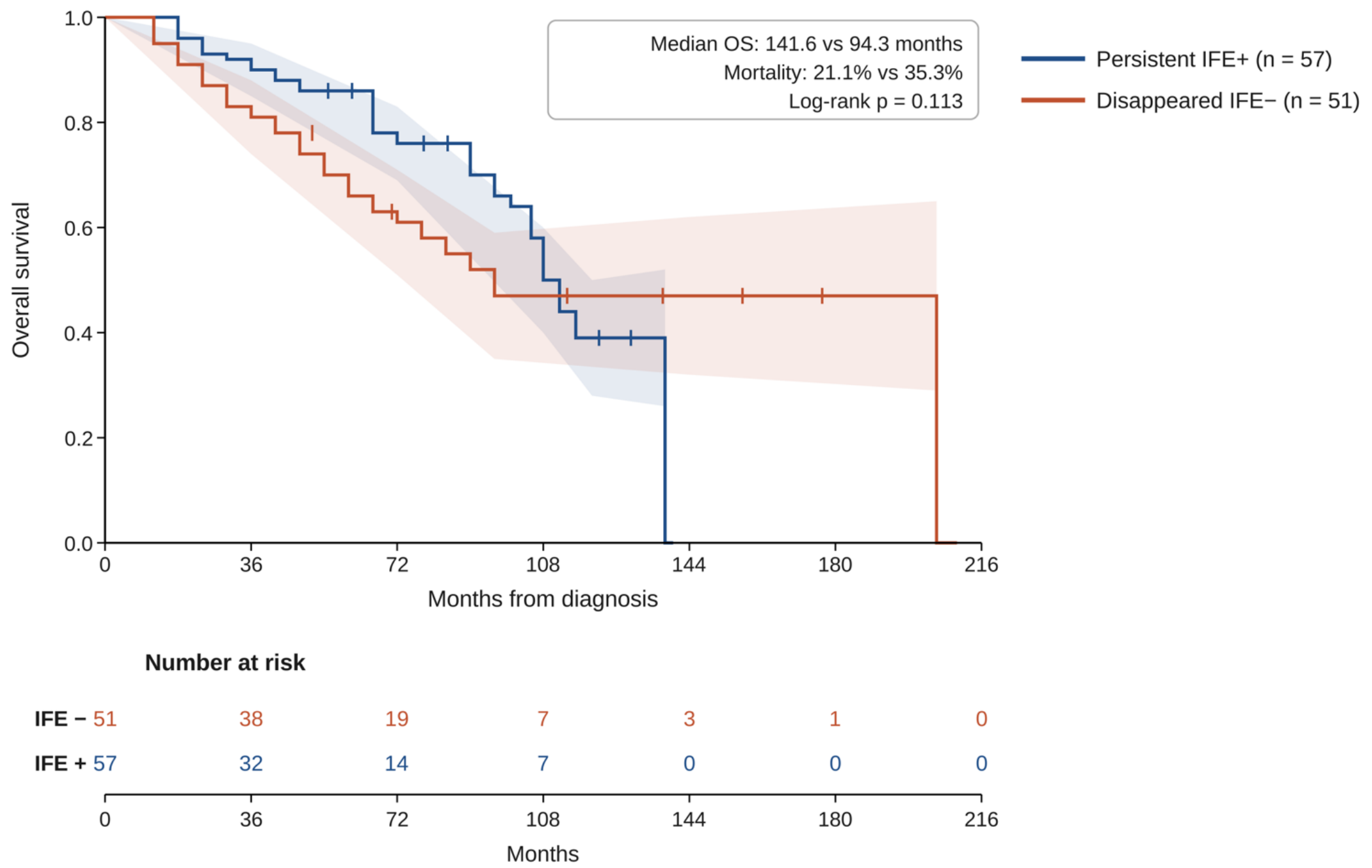

Supplementary Fig. S8. Overall survival according to on-treatment IFE status: Kaplan–Meier curves. Tick marks denote censored observations; shaded areas represent 95% confidence intervals. OS, overall survival; IFE, immunofixation electrophoresis.

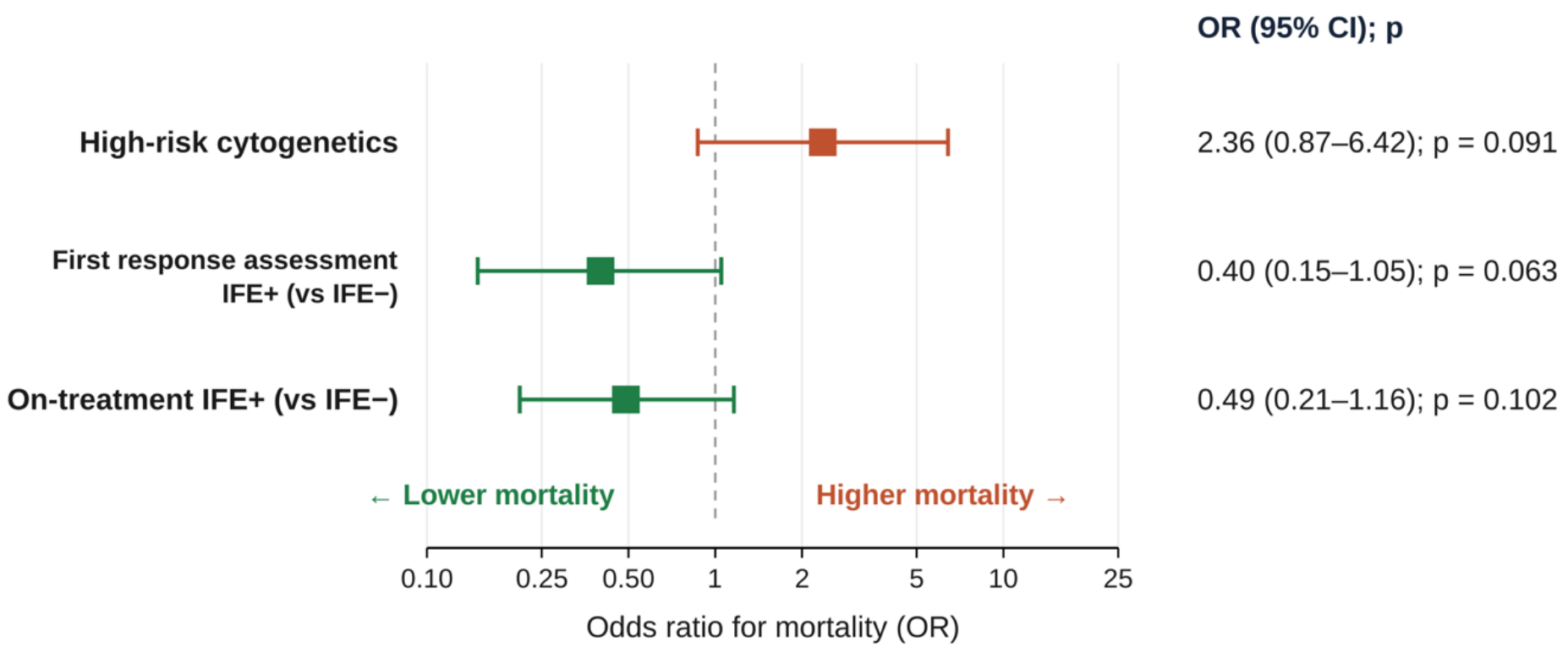

Supplementary Fig. S9. Factors associated with mortality: univariable analysis (logistic regression). Squares indicate the odds ratio and horizontal lines the 95% confidence interval; the dashed vertical line marks OR = 1. Arrows indicate the direction of the effect: left, lower mortality; right, higher mortality. OR, odds ratio; CI, confidence interval; IFE, immunofixation electrophoresis.
